# Mechanistic Insights into Dioxygen Transport Routes in the PHD2 Oxygenase from Long-Time Scale Simulations

**DOI:** 10.1021/acs.biochem.6c00039

**Published:** 2026-04-23

**Authors:** Brian Wiley, Simone Furini, Carmen Domene

**Affiliations:** † 1555 Departments of Chemistry University of Bath, Bath BA2 7AX, U.K.; ‡ Department of Electrical, Electronic and Information Engineering ‘Guglielmo Marconi’, University of Bologna, via Dell’Università 50, Cesena (FC) 47521, Italy

## Abstract

Understanding how dioxygen accesses buried catalytic centers in metalloenzymes is critical for elucidating enzymatic kinetics and guiding strategies to modulate catalytic activity. Here, we report over 20 μs of classical molecular dynamics simulations of the PHD2 oxygenase, a metalloenzyme regulating hypoxia signaling via HIF-1α hydroxylation. Our extended simulations reveal multiple dynamic dioxygen transport routes from solvent-exposed regions through the cupin fold to the metal active site, capturing transient interconverting channels and kinetic heterogeneity inaccessible to prior short-time scale studies. Dioxygen transport occurs on widely differing time scales, from rapid exchange (∼250 ps) to long residence times within internal hydrophobic cavities lasting hundreds of nanoseconds. These internal cavities act as dynamic reservoirs, modulating dioxygen availability and potentially contributing to the high K_m_ and slow oxidative turnover by PHD2. Analysis of cavity-lining residues identifies hydrophobic positions that may be targeted to tune catalytic rates. Collectively, our results refine the mechanistic model of dioxygen access in PHD2 and demonstrate how high-resolution simulations can uncover functionally relevant kinetic landscapes, providing principles applicable to the design and regulation of molecular catalysts.

## Graphical abstract

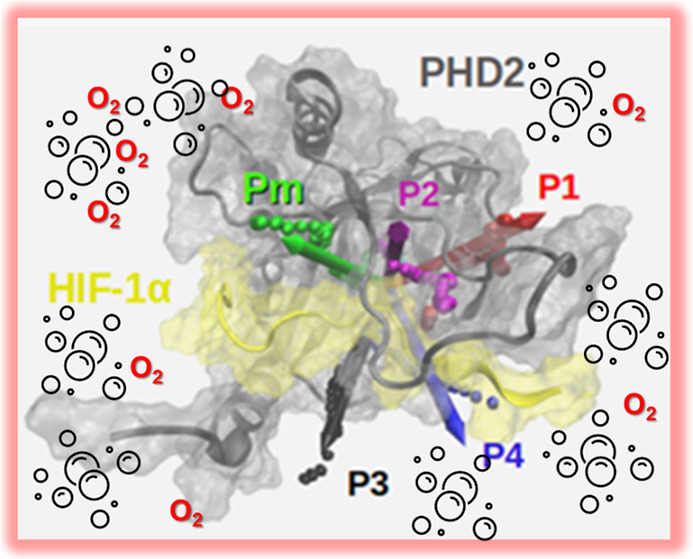

## Introduction

Oxygen is an essential gas for life on Earth. Since the great oxidation event (GOE), when cyanobacteria introduced dioxygen (O_2_) into Earth’s atmosphere approximately 2.4 billion years ago,^1^ enzymes have evolved to utilize oxygen for various biological processes. These include metabolism, for example, in cellular respiration where oxygen is essential for producing adenosine triphosphate (ATP), the cell’s primary energy currency, and regulatory mechanisms such as protein ubiquitination and targeted degradation. In biotechnology, oxygen-dependent enzymes have been cloned and expressed to catalyze the transformation of molecular scaffolds, serving as building blocks for new drugs such as viomycin, vancomycin, and β-lactams.^2,3^

There are many families of enzymes that utilize either one oxygen atom (monooxygenases) or two oxygen atoms (dioxygenases) to catalyze reactions that modify either their substrate alone or both the substrate and a cosubstrate/cofactor. These enzymes can be further classified based on the metal(s) they coordinate during catalysis, their cofactor dependency, and their structural protein folds. Common categories include cofactor dependent enzymes such as α-ketoglutarate-dependent oxygenases and heme-containing enzymes, as well as cofactor independent oxygenases. Additional distinctions include types such as mononuclear iron enzymes and ferredoxin/Rieske-type oxygenases, as well as structural fold families like cupins and α/β-hydrolases. A prominent representative of the Fe^2+^/α-ketoglutarate-dependent (AKG) dioxygenases is Prolyl Hydroxylase Domain-containing Protein 2 (PHD2), which regulates the hypoxic response via hydroxylation of Hypoxia-Inducible Factors (HIFs). HIFs are transcription factors that regulate the expression of numerous genes involved in cellular adaptation to low-oxygen (hypoxic) environments. PHD2 is one of three prolyl hydroxylases known to hydroxylate specific proline residues in HIF proteins.^4–6^ PHD1 and PHD3 share a similar domain architecture with PHD2 and catalyze hydroxylation at the C-4 position of proline residues. The PHD enzymes target conserved oxygen-dependent degradation (ODD) domains located at both the N-terminal (NODD) and C-terminal (CODD) regions of HIFs, specifically at LXXLAP motifs.^6^ Notably, PHD3 has not been shown to hydroxylate prolines within the NODD of HIFs, while PHD1 and PHD2 exhibit a preference for hydroxylating the CODD region.^7^

The fold surrounding the active site of the Fe^2+^/AKG-dependent dioxygenase family of enzymes consists of a double-stranded beta-sheet with a possible jelly roll/cupin (a small barrel) superfamily fold and aspartate-2-histidine (HXD···H) metal-binding coordination comotifs^7^ (see Figure 1). Early kinetic experimental methods for determining the rate of catalysis by PHDs and other Fe^2+^/AKG-dependent dioxygenases used simple decarboxylation assays by adding dioxygen and ascorbate to the PHD-HIF system and measuring the rate of radiolabeled ^14^CO_2_ production when one of the oxygen atoms is inserted into succinate, the product derived from AKG.^8^ More recent techniques, such as matrix-assisted laser desorption/ionization time-of-flight (MALDI-TOF) and surface plasmon resonance (SPR) have been used to measure both hydroxylated ODDs of HIFs and succinate levels, as well as binding affinities and rates of association/dissociation.^7,9^

**Figure 1:**
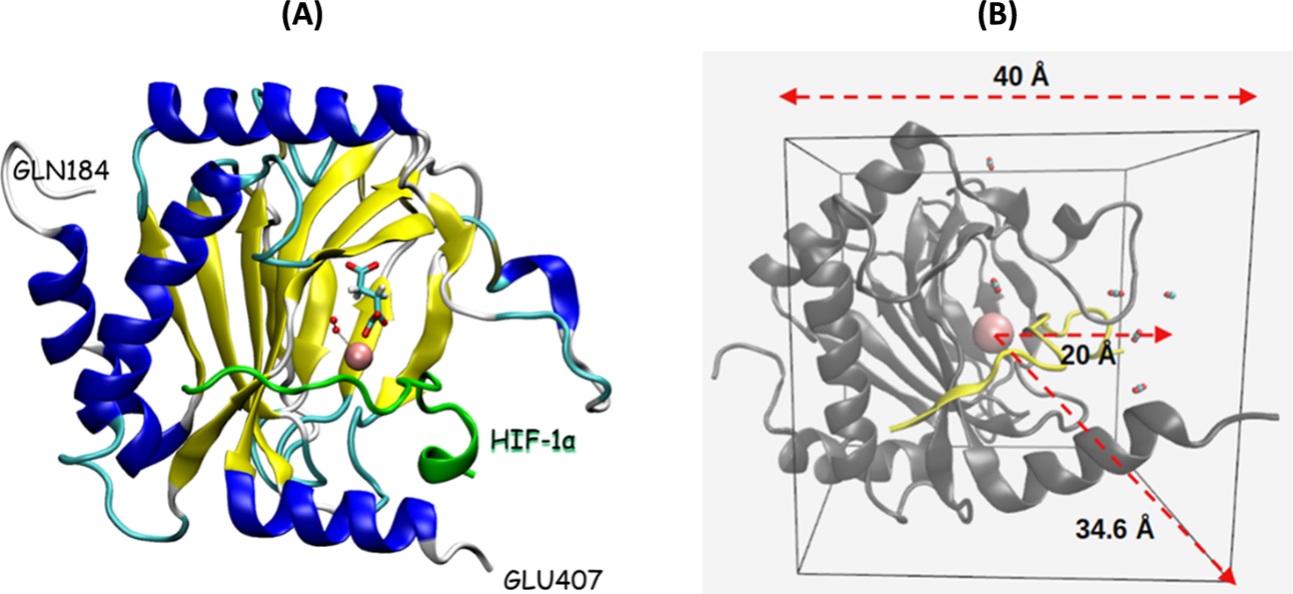
(A) Representative structure of PHD2 (PDB ID: 3HQR) colored by secondary structure, with HIF-1α shown in green, 2OG in licorice representation, Fe^2+^ shown in van der Waals representation in pink and dioxygen shown in red. (B) Representative MSM boundary state-space cell containing a central Fe^2+^ ion in pink, the protein in gray, HIF-1α in yellow and some dioxygen molecules in CPK representation.

Despite many interesting studies characterizing the activity of PHDs, little has been reported about the mechanisms of dioxygen entry into the catalytic site from an atomistic perspective.^10,11^ With the current power of high-performance computing and the widespread use of atomistic molecular dynamics (MD) simulations, we are well placed to investigate how dioxygen reaches its catalytic site in mono- and dioxygenase enzymes. Computational studies reported to date have employed the implicit ligand sampling (ILS) technique,^12^ used as a postprocessing method for MD simulations to construct one-to three-dimensional (3D) free energy landscapes or estimate energy barriers between energy minima, i.e., along pathways connecting cavities that harbor the gas molecules.^13–15^ Another technique employed, locally enhanced sampling (LES), utilizes multiple noninteracting copies of the same molecule that shares the same spatial region and interact with the rest of the system via averaged forces, thereby accelerating conformational sampling.^16,17^ These methods have been instrumental in identifying potential oxygen transport routes in several oxygen-dependent enzymes. Collectively, these results highlight the critical role of protein dynamics in modulating gas transport and catalysis.

In this respect, to date, atomistic studies of access tunnels in PHD2 have concentrated either on a single established transport pathway or on the proposed activation of the β2β3 loop.^18,19^ Notably, one study offered the first kinetic validation of this pathway using CAVER,^20^ a commonly employed software for mapping protein tunnels, thereby confirming a primary hydrophobic route to the PHD2 active site.^10^ Here, leveraging extended-time scale MD simulations and two complementary force fields for dioxygen, our goal is to provide new insights into how varying dioxygen concentrations and internal hydrophobic networks influence the formation and persistence of alternative “cavity-hopping” tunnels that may regulate access to the buried catalytic site.

## Methods

### System Setup

The starting model (PDB ID: 3HQR)^21^ was the structure of PHD2 in complex with Mn^2+^ as a cofactor, N-oxalylglycine, and the hypoxia-inducible factor 1-alpha (HIF-1α) C-terminal oxygen-dependent degradation domain (CODD) residues 558–574 (UniProt Q16665), which contains a single hydroxylated proline residue (Pro564). Mn^2+^ was replaced with Fe^2+^, and N-oxalylglycine was substituted with the natural cosubstrate 2-oxoglutarate (2OG), also known as α-ketoglutarate (AKG) (see Figure 1).

The protein and ions were modeled using the CHARMM force-field.^22^ Parameters for 2OG were taken from ref 10, where they were originally developed (see Supporting Information of that work for details). Parameters for Fe^2+^ cations compatible with the CHARMM additive force field and the TIP3P water model were taken from ref 23. The system was solvated with TIP3P water molecules^24^ and sodium ions were added to neutralize the system. Randomly selected water molecules were replaced with dioxygen molecules. Two force field parameters were employed for dioxygen, the O_2_IF^25^ and the O_2_QD^26,27^ models. The simulation box dimensions were approximately 80 × 80 × 80 A. The final simulation systems had over 50,000 atoms.

Systems containing 25, 50, or 100 dioxygen molecules were simulated with 10 replicates each, corresponding to approximate concentrations of 0.09, 0.18, and 0.37 M, respectively. These elevated concentrations were intentionally chosen to enhance statistical sampling of dioxygen entry into PHD2 and do not reflect the millimolar oxygen levels typically present under physiological conditions. Interactions between dioxygen molecules were assumed to have a negligible effect on gas transport dynamics, consistent with previous studies.^10^

The system was energy-minimized using the steepest descent integrator to a maximum force tolerance of 1000 kJ mol^–1^ nm^–1^ and equilibrated using a decreasing position restraint schedule on the protein backbone and side-chain atoms for a total of 500 ps under the NVT ensemble, followed by NPT equilibration for 250 ps. Temperature coupling was performed using the Nosé–Hoover thermostat.^28^ Long-range electrostatics were modeled using the fast smooth Particle-Mesh Ewald (SPME) method.^29^ The Verlet cutoff scheme^30^ was used for neighbor searching, and the LINCS algorithm^31^ was applied to constrain hydrogen bonds. Production runs were performed using a 2-fs time step and the leapfrog integrator to solve Newton’s equations of motion at the NPT ensemble using the Parrinello–Rahman barostat.^32^ All simulations were conducted with GROMACS version 2020.3,^33^ and they are described in Table 1.

**Table 1:** Summary of the Simulations Employed in This Study

model	# O_2_ molecules	# replicas	total time (μs)
O_2_IF	25	10	10 μs
	50	10	10 μs
	100	10	10 μs
O_2_QD	100	20	18 μs

For the MD analysis, configurations were sampled at 10 ps intervals, applying a cutoff based on the center-of-mass (COM) distance between dioxygen and Fe^2+^ of 6.0 Å. To identify dioxygen access to the active site, we monitored time points during the simulation when any dioxygen molecule approached the metal center within 6 Å. Although prior studies have suggested that catalytically productive binding typically requires dioxygen to be within 4–5 Å of the iron center,^10,34^ a 6 Å threshold was selected to include potential approaches and entry events.

For each instance in which a dioxygen molecule reached this threshold, its trajectory was traced both backward and forward in time to characterize entry and exit pathways. Trajectories were followed until the dioxygen molecule reached 20–25 Å from the iron center, which encompasses the full volume of PHD2 relevant to substrate access. This strategy allowed us to focus on key events while maintaining a temporal resolution of 10 ps.

### Markov State Models

Markov State Models (MSMs) were constructed to analyze dioxygen-access pathways based on the simulation trajectories. Individual dioxygen positions were extracted at 100 ps intervals from each simulation across all experiments. All frames were superimposed and then, for each frame, the position of each dioxygen molecule was subtracted from the Fe^2+^ coordinates to express positions relative to the catalytic center. These relative coordinates were then binned onto a 3D grid spanning the periodic boundaries of the solvated system with grid spacing of 1 Å in each dimension. A cubic cutoff of 40 Å centered on Fe^2+^ was applied, such that any molecules positioned outside this cutoff were wrapped to the corresponding boundary of the simulation box along the *X*, *Y*, and *Z* axes. Discrete trajectories of each dioxygen molecule were constructed based on their positions within the defined grid over time. Each grid location within the simulation box was treated as a distinct state in the MSM.

The set of possible states, *N*, corresponds to all grid points occupied by dioxygen molecules, with a theoretical maximum of 64,000 states (40^3^ grid points). The transition probability matrix, ***T*** (τ), in the MSM is explicitly defined as the probability that the system is in state *j* at time *t*+τ, given that it was in state *i* at time *t* ^35^

$$T_{ij}\left(\tau \right)=P\left(X_{t+\tau }=j\;|\;X_{t}=i\right)$$

where *X*_t_ is the discrete state of a dioxygen molecule at time *t, i, j* ∈ 1, ..., *N* are the grid-based states in the simulation box, *N* is the number of observed states after filtering, and τ is the chosen lag-time. States that were never visited or had no observed transitions were excluded from *T*(τ), and each row was normalized such that ∑_j_ *T*_*ij*_ (τ) = 1.

The Maximum Likelihood Estimator (MLE) for the MSM was constructed by maximizing the likelihood of observing the discrete trajectories,^36^ given the transition probabilities

$$L\left(T\right)=\prod_{i,j}{\left[T_{ij}\left(\tau \right)\right]}^{c_{ij}}$$

where *c*_*ij*_ is the number of observed transitions from state *i* to *j* at lag-time τ. This procedure was implemented using the PyEMMA^37^ and Deeptime Python^38^ packages. Bayesian MSMs were additionally estimated by sampling the posterior over transition matrices given the same observed transitions. Lag-times were systematically tested (1–100 × 100 ps) and models were evaluated via implied time scales and Chapman–Kolmogorov tests to ensure Markovianity.

For the MSM analysis, frames were sampled at 100 ps intervals, and the COM distances were shifted to the nearest grid point. Using a cutoff of 6.7 Å in the MSM yields a similar number of events as the 6.0 Å cutoff used in the MD analysis. Note that the correspondence between the MSM and MD analyses is not strictly one-to-one because the MSM uses frames sampled every 100 ps, whereas the MD analysis is based on the original trajectory saved every 10 ps, and the MSM grid adjustment can shift *a* point to slightly above or below 6.7 Å, depending on its position relative to the nearest grid point.

While the MSM-derived mean-first-passage times provide a systematic framework for analyzing O_2_ migration, we note that discrepancies between direct MD trajectories and MSM results can arise from statistical sampling limitations and the filtering procedure used to define event states. Deviations from linearity in the mean squared displacement (MSD) of O_2_ indicate subdiffusive behavior (α < 1), which can affect the time scales observed in MSMs compared to raw MD analysis. These caveats highlight that MSMs capture the dominant pathways and overall kinetics, but small-scale fluctuations or rare events present in MD trajectories may not be fully represented. We therefore interpret MSM results in the context of these limitations and the observed subdiffusive trends across pathways.

To validate the Markovian assumption of these models, we performed Chapman–Kolmogorov tests, which compare the predicted probabilities of transitioning between states over multiple lag times (obtained by iteratively multiplying the MSM transition matrix) with the observed transition probabilities directly measured from the trajectories. Good agreement between predicted and observed probabilities indicates that the MSM captures the system dynamics accurately and behaves approximately as a Markov process at the chosen lag times.

A representative MSM boundary state-space cell is illustrated in Figure 1 to clarify the spatial definition used in the model. Event states were defined as those in which the radius from the dioxygen grid point to the catalytic Fe^2+^ was less than 6.7 Å, a threshold slightly greater than the 6 Å criterion to account for mapping to the nearest grid point. For inward events, trajectories were traced backward to the last frame at which the dioxygen grid point exceeded 22 Å from Fe^2+^; for outward events, forward tracing was similarly performed. Each state along the trajectory was recorded. The original set of grid points and their distances to Fe^2+^ were subsequently filtered, and any state not included in this filtered set was treated as a null state. MLE MSMs were then constructed using lag times of 0.1, 0.2, 0.5, 1.0, 2.0, and 5.0 ns.

Reduced-state MSMs were used exclusively for Bayesian inference and posthoc validation of Markovianity. These reduced models were constructed by filtering for event states located within 7 Å of the Fe^2+^ catalytic center and using a milestoning procedure to account for intermediate and null states along trajectories. This reduction was necessary for Bayesian inference and Chapman–Kolmogorov testing in order to reduce the computational cost related to eigenvalue and eigenvector decompositions.

Importantly, the MFPTs reported in this work were computed using the maximum-likelihood MSM constructed over all active states and do not rely on this state-space reduction. We verified that MFPTs obtained from reduced and full-state models are statistically consistent.

## Results

### Backward and Forward Tracking of Dioxygen Trajectories to and from the Catalytic Site

Translocation events, both inward and outward, were first identified by tracking frames in which any dioxygen molecule reached within 6 Å of the catalytic site, defined as proximity to the Fe^2+^ center. This threshold was selected based on a survey of the literature and typical distances between dioxygen and the coordinating HXD···H motif.^12^ From this minimum distance, each trajectory was traced backward and forward in time to identify the last frame before inward approach and the first frame after outward departure in which the position of the dioxygen molecule exceeded 20 Å from the Fe^2+^ center. The corresponding trajectory segments were then examined manually to analyze each event (Figure 2). A total list of paths, residence times, and atomic contacts associated with dioxygen motion was compiled using custom Python scripts developed with the MDTraj package.^39^ This “manual” verification was essential, as surface distances from solvent to the active site varied between ∼11 and ∼18 Å depending on the specific trajectory. This variability required detailed inspection to validate and refine path assignments.

**Figure 2:**
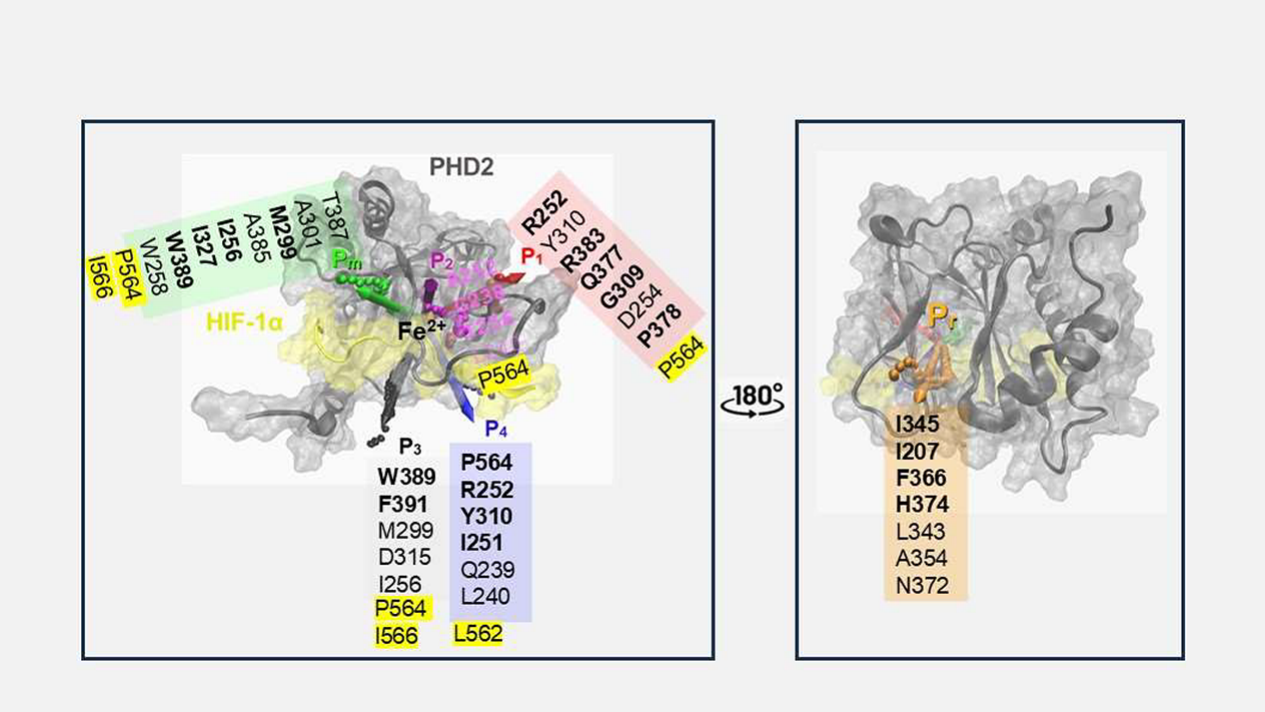
Six distinct pathways for dioxygen access to the catalytic Fe^2+^center in PHD2. Schematic representation of all six dioxygen pathways, annotated as P_1_ (red), P_2_ (pink), P_3_ (black), P_4_ (purple), P_m_ (green) and P_r_ (orange), with their most frequently contacted residues. Residues from HIF-1α are highlighted in yellow.

Analysis of the simulations identified a total of 642 events into and out of PHD2 across six distinct pathways (Table 2; Figure 2). The number of events was strongly dependent on dioxygen concentration. Simulations containing only 25 or 50 dioxygen molecules yielded just 47 and 50 events, respectively, whereas increasing the concentration to 100 molecules resulted in a dramatic rise: 249 events with the O_2_IF force field and 298 events with the O_2_QD force field. Averaged over the 100-molecule systems, these correspond to approximately 25 events per μs (one event every 40 ns) for O_2_IF and 17 events per μs (one event every 59 ns) for O_2_QD (Tables 2 and 3), highlighting the pronounced effect of dioxygen availability on entry into PHD2.

**Table 2:** Frequencies of Dioxygen Entries into the Different Pathways^a^

model # molecules	events (μs)/total # events	P_m_ # events entry/exit	P_r_ # events entry/exit	P_4_ # events entry/exit	P_3_ # events entry/exit	P_2_ # events entry/exit	P_1_ # events entry/exit
**O**_**2**_ **IF 25**	4.7/47	**26**/23	**8**/8	**7**/4	**5**/3	**3**/3	**3**/1
**O**_**2**_ **IF 50**	5/50	**27**/31	**6**/6	**4**/5	**2**/2	**6**/3	**5**/3
**O**_**2**_ **IF 100**	25/249	**66**/66	**98**/98	**55**/54	**11**/10	**6**/11	**13**/10
**O**_**2**_ **QD 100**	17/296	**158**/147	**34**/34	**59**/47	**30**/23	**18**/25	**9**/8

a For each pathway, the number of entry/exit events, measured from 20 Å to <6 Å and back, is reported. Labels for the pathways are P_m_ = main path, P_r_ = reverse β-sandwich entry, P_1_ = between the β2β3 loop and the first β-strand (cis-HIF-1α side), P_2_ = through the β2β3 loop, P_3_ = under HIF-1α, P_4_ = across the HIF-1α CODD N-terminus when the PHD2 loop opens toward the PHD2 N-terminus. Frames employed were those saved at 10 ps intervals.

**Table 3:** Characteristic Times Associated with Dioxygen Movement Along Pathways^a^

	$\overset{®}{t_{entry}}$ | $\overset{®}{t_{exit}}$ (ns)					
model # molecules	**P**_**m**_	**P**_**r**_	**P**_**4**_	**P**_**3**_	**P**_**2**_	**P**_**1**_
**O**_**2**_ **IF 25**	5.4 | 3.5	4.0 | 2.7	2.4 | 2.1	46.5 | 3.0	12.6 | 12.0	2.5 | 3.3
**O**_**2**_ **IF 50**	9.4 | 6.4	3.5 | 5.7	3.6 | 4.5	3.3 **| 0.2**	5.8 | 5.0	5.9 | 8.7
**O**_**2**_ **IF 100**	11.5 | 9.6	1.8 **| 1.4**	0.8 | 1.9	1.0 | 2.8	32.9 | 7.2	7.5 | 7.9
**O**_**2**_ **QD 100**	6.0 | 7.3	1.5 **| 1.4**	1.5 | 2.5	2.0 | 1.8	4.9 | 4.3	1.8 | 15.2

a For each pathway, the average entry and exit times in nanoseconds, measured from 20 Å to <6 Å from Fe^2+^ and back, are reported. Pathway labels are as in Table 2. Frames employed were those saved at 10 ps intervals.

Analysis of the pathways revealed that three, P_1_, P_2_, and P_3_, accounted for less than 20% of all translocation events combined, with each contributing under 7.5% (Table 2). Despite their low frequency, these pathways provide distinct routes to the catalytic site. The least populated pathway, P_1_, approaches the catalytic site from the left of the final β-strand on the HIF-1α side, threading between the β2β3 loop and the cupin barrel (Figures 2,3,4). Pathway P_2_ enters directly through the β2β3 loop between Gly238 and Asp254 (Figure 2, magenta-colored loop and surface), whereas P_3_ passes beneath the HIF-1α CODD, traveling between the peptide and PHD2 toward the catalytic residues Asp315 and His314. The third most common path, P_4_, emerges when conformational changes occur in either the β2β3 loop, residues Gly238–Asp254, previously described by Flashman et al.^7^ and/or the first few helical residues of HIF-1α CODD (Asp558–Leu562; yellow helix in Figure 2). This helix was also observed to unwind during simulations, transiently forming a tunnel from solvent to the catalytic metal ion (Figure 2). The second most frequent pathway, P reverse (P_r_), allows entry from the opposite side of the cupin fold, and is therefore referred to as the anti-PHD2-HIF-1α or reverse β-sandwich pathway. Dioxygen accesses the catalytic site via a range of subpaths along the β-sheets from this side (Figure 3). Finally, the dominant pathway, P main (P_m_), accounted for nearly half of all translocation events. This path, frequently reported in the literature, involves entry through the top of the cupin barrel when viewed from the HIF-1α CODD and the C-terminal helix of PHD2. It forms a broad “vortex” of entry points centered above the HIF-1α peptide and is partially gated by the β2β3 loop, depending on its conformation.^19,40^ This route, often referred to as the “active site lumen”, has been associated with thermal stability of PHD2 and can be considered the cis-PHD2/HIF-1α pathway since it lies on the same side of the cupin fold as the substrate.

**Figure 3:**
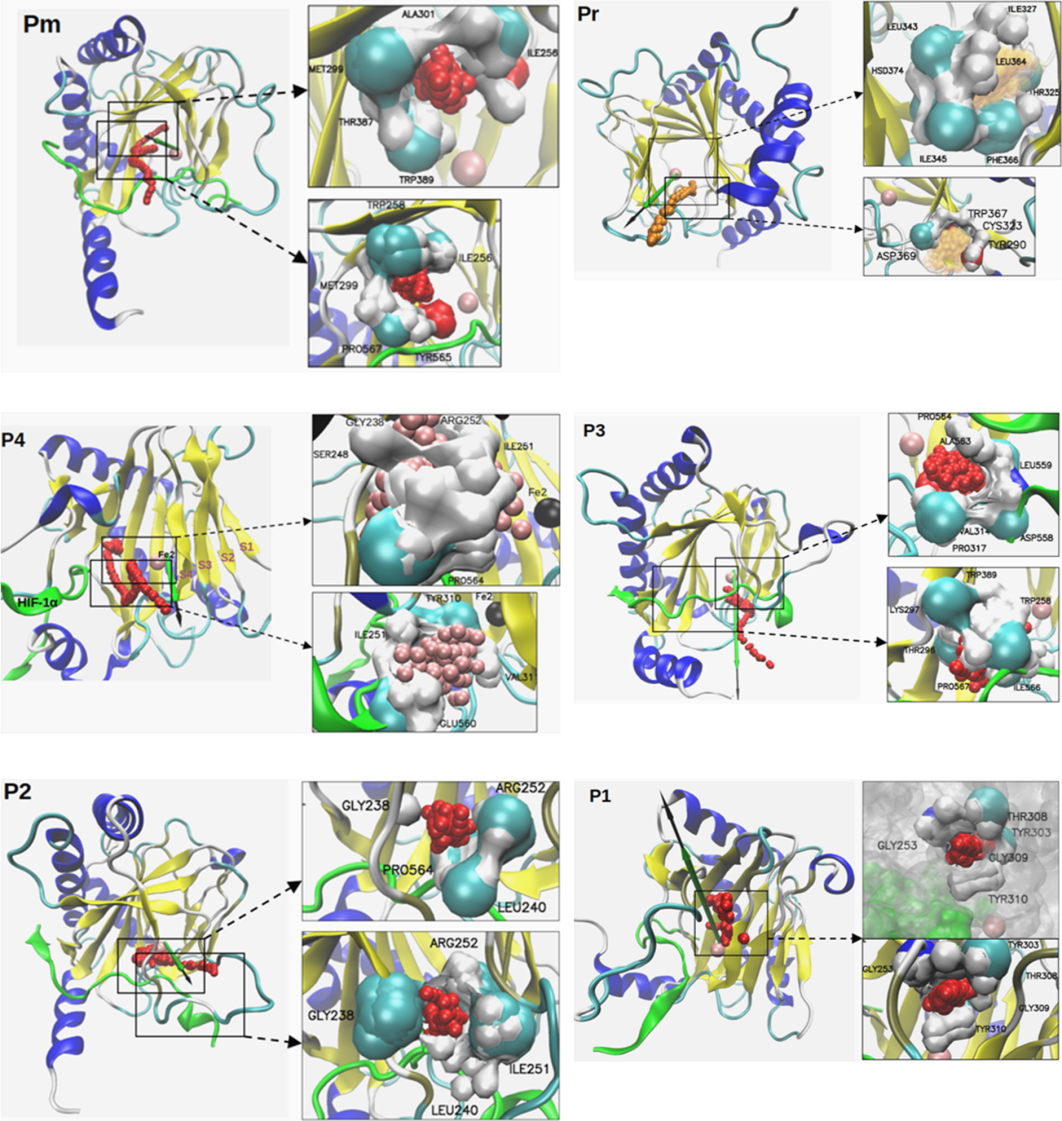
Hydrophobic cavities along the six identified access pathways form transient tunnels aligned with the PHD2 catalytic site. A vector extends from Fe^2+^ to the protein surface, illustrating an example dioxygen trajectory (small red sphere). PHD2 is in cartoon representation colored by secondary structure, and HIF-1α in green cartoon. Arrows indicate radial distance from Fe^2+^: green = 20 Å, black = 25 Å. Highlighted regions mark representative hydrophobic cavities where dioxygen resides temporarily (500 ps to 10 ns, up to ∼400 ns). Small spheres show dioxygen positions over time, with transparency reflecting shorter residence times or smaller cavities. Hydrophobic interactions are shown for selected frames (10–30 ps), highlighting side-chain atoms within 3.5 Å of dioxygen. Atoms are colored by element: H (white), C (cyan), O (red), N (blue).

**Figure 4:**
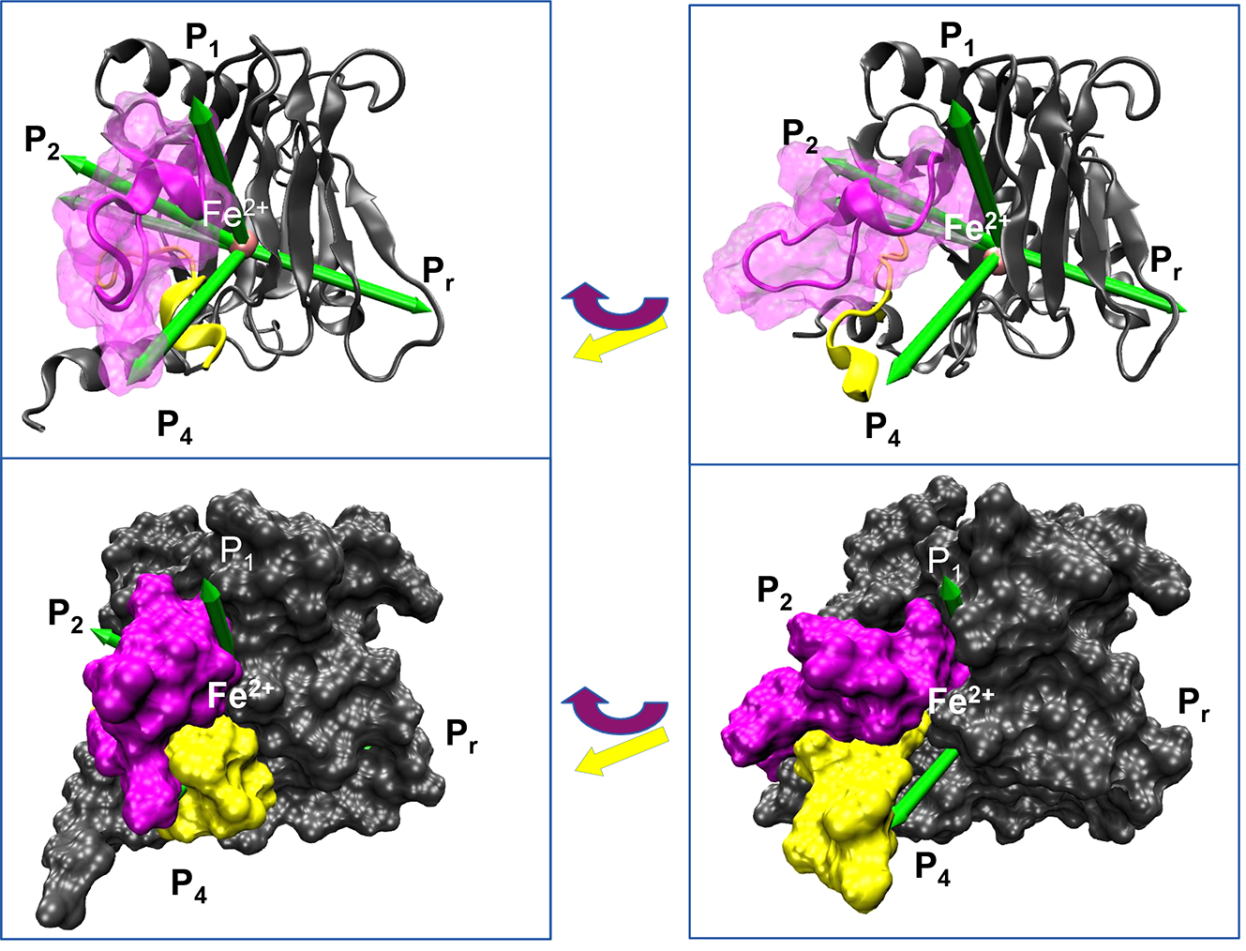
Conformational dynamics of the β2β3 loop in PHD2 (magenta) relative to the HIF-1α CODD peptide (yellow) reveal the transient formation of a novel pathway. P_4_ is short-lived, reflecting the flexibility of both the β2β3 loop and the peptide. Representative frames are shown at 0 and 1 μs (left and right respectively). Two protein representations are included for clarity. The arrows in the center illustrate the motion of the β2β3 loop (magenta) and the HIF-1α CODD peptide (yellow), indicating that either both move together, or the loop moves first, allowing the small helix at the end of HIF-1α CODD to partially unwind and permit dioxygen entry.

Across all simulation sets, regardless of the dioxygen force field used, we observed consistent pathway characteristics, including similar pathway proportions, average minimum distances between dioxygen and the catalytic Fe^2+^ ion, and inward and outward translocation times (Table 3).

The primary pathway, P_m_, did not account for the highest number of events in the O_2_IF simulation with 100 dioxygen molecules (Table 4) largely due to a single outlier replicate in which 92 events (inward and outward) occurred via the reverse pathway (P_r_) with just over 1 μs of simulation time, that is, approximately one event every 10 ns. Across simulations, the average minimum distance to Fe^2+^ ranged between 4.6 and 5.5 Å. The P_4_ pathway exhibited the fastest inward times, 0.8 ns, in the simulations with 100 dioxygen molecules using the O_2_IF model. In contrast, the P_3_ pathway showed the fastest outward times in simulations with 50 dioxygen molecules and both force fields employed (Table 3). If residence time near the active site correlates with catalytic probability, these data suggest that the P_r_ pathway may be least favorable for productive catalysis based on the outward diffusion time for P_r_ in Table 3. Interestingly, dioxygen does not always exit through the same pathway by which it entered. The only consistent exception was observed for dioxygen molecules entering from the anti-HIF-1α side (P_r_), which may reflect geometric or steric constraints imposed by the narrower tunnel of the reverse side of the cupin fold, potentially enhancing directional “squeezing” of dioxygen out of the active site.

**Table 4:** Simultaneous Events and Residue Contacts **d**uring Dioxygen Acces**s** ^a^

	P_m_	P_r_	P_4_	P_3_	P_2_	P_1_
**total in**	277	146	125	46	42	30
**total out**	267	146	110	40	33	22
**% exit** **via same path as entry**	83	100	78	37	40	50

number of simultaneous access events across pathways						
	**P**_**m**_	**P**_**r**_	**P**_**4**_	**P**_**3**_	**P**_**2**_	**P**_**1**_
**P**_**m**_	125	10	24	13	19	5
**P**_**r**_		61	0	0	0	0
**P**_**4**_			18	11	2	7
**P**_**3**_				5	2	5
**P**_**2**_					5	3
**P**_**1**_						7

a This analysis includes cases where dioxygen molecules become transiently trapped, remaining in contact with one or a few residues for 75–90% of the total access time. Columns report: total access time for entry (total time in, ns) and exit (total time out, ns); the percentage of trajectories exiting via the same path as entry (% exit via same path as entry); the number of simultaneous access events across pathways; and the fraction of contacts with key residues or regions (P_m_, P_r_, P_4_–P_1_) along the access pathways.

Another notable observation is the frequency of overlapping or simultaneous access events within and between pathways (Table 4). The P_m_ pathway exhibited the highest number of overlapping events, both within the same pathway (125 intrapathway overlaps) and with other routes. Specifically, P_m_ showed strong overlap with P_4_ (61 events) and, to a lesser extent, with P_r_ (10). This dominance suggests that P_m_ represents the principal conduit for dioxygen migration, acting as a central or “hub” pathway dynamically connected to neighboring routes. The relatively high overlap between P_m_ and P_4_ implies that these two channels may share partially overlapping entry or transition regions, or that dioxygen molecules can transiently interchange between them along the access pathways.

In contrast, the peripheral pathways, particularly P_2_ and P_1_, displayed very few overlaps, both within the same pathway and with other routes, indicating that these channels are more isolated or infrequently sampled by dioxygen. Intermediate levels of overlap among P_r_, P_4_, and P_3_ (ranging from 11 to 18 events) suggest moderate crosstalk or transient connectivity between these secondary access routes, possibly reflecting local flexibility or breathing motions within the protein matrix.

These observations refine the earlier view that dioxygen accesses the PHD2 active site through a single dominant hydrophobic tunnel. Instead, the present data indicate a more complex transport network comprising multiple, partially interconnected channels. The predominance of events within individual pathways, combined with selective interpathway overlaps, supports a model in which dioxygen molecules primarily follow preferred routes (such as P_m_) but can occasionally transition between adjacent pathways. This behavior points toward a degree of cooperative or concerted access dynamics, facilitating efficient dioxygen delivery to the buried catalytic site despite the restricted solvent accessibility of the enzyme’s active center.

### PHD2 and HIF-1α-CODD Residue Contacts along Dioxygen Pathways

The most frequently observed pathway in PHD2, P_m_, is formed by a vortex of residues at the cis-PHD2/HIF-1α interface. These residues possess either large, predominantly hydrophobic side chains or amphiphilic character, with polar or partially negatively charged atoms oriented away from dioxygen. This arrangement likely facilitates hydrophobic tunnelling of dioxygen. The prevailing hypothesis in the field is that gases access the active site in enzymes such as oxygenases through transient hydrophobic channels that open and close dynamically, forming a network of linked cavities.^15–17,35,37^ In line with this, numerous studies ranging from time-resolved X-ray crystallography to classical MD simulations, have been used to identify internal cavities in gas-converting proteins.^10,34,37,38^

In the P_m_ pathway, the initial hydrophobic cavity closest to the PHD2-HIF-1α interface (Figure 3) is relatively short-lived, which is consistent with its proximity to the protein surface and solvent interface. This cavity is composed of residues such as Pro567, Ile566, the aromatic ring of Tyr565, with the hydroxyl group oriented away from dioxygen toward Pro564, Trp258, Ile256, and the terminal methyl group of Met299. As dioxygen migrates deeper into the PHD2 cupin fold, it reaches a longer-lived hydrophobic cavity (Figure 3) that includes Met299 and Ile256, as well as Trp389, the terminal methyl of Thr387, Ala301, Ala385, and Ile327 (Table 5). This cavity lies on average ∼8 Å from Fe^2+^, with dioxygen observed to briefly approach as close as ∼5.1 Å on average before returning to the cavity.

**Table 5:** Residue Contact Frequencies for Dioxygen^a^

	P_m_	P_r_	P_1_	P_2_	P_3_	P_4_
in	T387 44.39	I345 61.15	Y303 39.52	R252 43.05	R322 76.00	P564 48.45
	A301 43.96	I207 51.89	V376 38.96	M299 40.31	I566 73.79	Y310 35.26
	M299 43.74	F366 43.93	Y310 26.10	P564 38.28	F391 59.45	H313 32.19
	A385 34.87	L343 27.92	P564 22.94	I256 35.20	T296 57.79	R252 28.87
	I566 29.82	N37 27.24	R252 20.92	T387 34.93	W389 43.56	I251 28.04
	I256 28.78	H374 22.16	D254 19.70	A301 32.65	R396 42.49	A563 25.68
	I327 23.25	A354 19.80	R383 15.67	G238 31.10	P564 15.74	L559 19.80
	W389 22.80	P347 16.98	M299 15.25	W389 27.22	D320 12.38	L562 19.37
	P564 21.99	I356 15.57	G309 15.16	A385 19.09		L240 19.15
		I327 14.36	I566 13.22	Q239 16.96		V311 17.15
in**	T387 14.87	I345 35.59	Y310 19.46	R252 36.49		P564 28.31
	A301 15.76	I207 36.69		M299 38.44		Y310 19.47
	M299 37.75	F366 28.09		T387 29.49		H313 11.44
	I566 17.11	L343 20.28		G238 27.46		R252 24.28
	I256 21.86	N372 21.22		P564 20.71		I251 15.30
	P564 16.53					L240 14.14
	W258 15.92					V311 13.08
						Q239 12.32
out	M299 48.55	I345 57.53	V376 70.79	**P564** 66.80	**I566** 54.62	**P564** 36.16
	T387 48.04	I207 48.93	Y303 65.76	G238 51.46	R322 44.42	I256 30.57
	A301 44.07	F366 43.26	A379 47.95	R252 48.89	W389 35.93	R252 30.48
	A385 37.59	L343 27.81	G341 46.72	I256 40.95	T296 33.06	M299 27.11
	**I566** 30.39	N372 27.34	R383 43.50	M299 33.54	F391 27.66	A301 22.08
	W389 26.80	H374 22.96	Q377 41.83	L240 32.69	**P564** 25.31	T387 21.48
	I327 25.08	A354 17.73	G309 28.09	Q239 26.49	R396 16.44	H313 16.64
	I256 24.76	I356 15.04	P378 19.08	W258 23.60	M299 15.09	I251 16.38
	**P564** 23.53	L364 14.42	R252 17.35	**I566** 23.25		G238 16.31
	W258 21.60	P347 14.11	D254 16.91	I251 18.76		Y310 15.85
out**	M299 34.76	I345 34.80	R252 14.20	**P564** 35.32	**I566** 18.16 R322 10.56	I256 25.26
	W389 24.14	I207 35.06	Y310 14.59	G238 41.49	**P564** 14.58	
	I327 20.16	F366 32.70				
	I256 19.21	L343 15.18				
	P564 15.00	N372 19.60				
	W258 16.14					
	**I566** 17.86					
	R252 17.76					
	I386 13.50					
	Q239 13.24					

a Residue contacts are reported for the ten residues with the highest contact frequencies. A contact is defined as any atom of a residue being within 3.5 Å of either oxygen atom in dioxygen. Contact frequency is expressed as contacts per nanosecond. “In/Out” analysis is also presented, which excludes cases in which dioxygen becomes transiently trapped, e.g., when dioxygen remains in contact with a single residue for >75% of the time; these data are labelled In** and Out**. In such cases, only that specific residue/time-in pair is removed from the dataset. Residues that are new relative to the previous analysis are shown in red. Residues from HIF-1α are highlighted in bold yellow. Only contacts above 12% are recorded.

In the P_r_ pathway, several smaller or short-lived hydrophobic cavities were also observed. As in P_m_, dioxygen progresses into a deeper cavity at the opposite end of the cupin barrel. One particularly notable hydrophobic cavity is located near the base of the first strand in the internal β-sheet of the cupin fold. In this region, the carboxylate of Asp369 points outward toward the solvent, while the aromatic rings of Trp367 and Tyr290 create a cavity favorable to dioxygen occupancy. The sulfhydryl group of Cys323 is typically oriented with the hydrogen facing dioxygen. This deeper cavity, which may be the closest hydrophobic pocket to the catalytic Fe^2+^ center, lies at an average distance of 4–6 Å (Figure 3). It includes catalytic triad residue His374, Leu343, Leu364, Ile327, Ile345, Phe366, and the terminal methyl of Thr325 (Table 5).

In a subset of replicates, a total of three out of ten for both the 50- and 100-O_2_ O_2_IF systems, and eight out of 20 for the 100-O_2_ O_2_QD system, a conformational fluctuation was observed in the PHD2 β2β3 loop (residues Gly238–Asp254). This fluctuation ranged from minor shifts to a complete swing of the loop and appeared to loosen the packing around the short α-helix of the HIF-1α CODD domain, occasionally unhinging it (Figure 4). Whether this fluctuation occurs naturally is uncertain, as the structure used lacked N-terminal residues prior to Asp558. No known kinetic studies have directly compared different CODD peptide lengths; only comparisons between NODD and CODD 19-mers or between extended constructs have been reported.^9,41–43^

This conformational change opens a surface-accessible route to Fe^2+^, which occasionally becomes almost fully exposed, allowing dioxygen to enter at multiple angles and potentially facilitating access for chelating water molecules.^44^ Favorable orientations of Leu559 and Met561 in HIF-1α further open this pathway, while Glu560 points away from the P_4_ path, toward the solvent (Figure 4). The initial cavity along the P_4_ pathway includes favorable hydrophobic interactions with Ile251, the aromatic ring of Tyr310, Val311, and the carboxylate of Glu560, which is oriented away from dioxygen. The deeper cavity lies directly above Pro564, the site of hydroxylation during catalysis, and is enclosed by residues in the PHD2 β2β3, which act as a protective cap over Pro564. These residues include Gly238, Leu240, Ser248 (with its hydroxyl group facing outward), Ile251, the aliphatic side chain of Arg252, and the methylene groups of the Pro564 pyrrolidine ring.

Pathway P_3_ traverses from directly beneath HIF-1α, when viewed from the cis-PHD2/HIF-1α interface. This pathway would likely support the fastest dioxygen translocation, were it not for a frequent detour. Dioxygen often diverts into a hydrophobic cavity that overlaps the deeper pockets of the P_m_ pathway (Figure 3), but lies slightly lower, closer to the CODD backbone. Residues contributing to this detour cavity include Ile566 and Pro567 (HIF-1α), as well as Trp258 and Trp389 (PHD2). Additional hydrophobic interactions are provided by the methylene groups of Lys297 and Thr296. This cavity lies approximately 8 Å from Fe^2+^, similar in distance to the deep P_m_ cavity, but shifted downward in the *XY*-plane. These detour cavities may transiently trap dioxygen, delaying access to the catalytic site. Before entering the detour, the initial cavity of P_3_ includes His313, a member of the catalytic triad, along with Val314 and Pro317 (PHD2), and residues Asp558, Leu559, Ala563, and Pro564 (HIF-1α).

The P_2_ pathway, which passes through the β2β3 loop, occurs less frequently than the adjacent P_4_ pathway but may still hold significance. Together with the P_1_ and P_4_ paths, it supports the hypothesis that differences in the β2β3 loop sequences among the three PHD isoforms contribute to distinct substrate specificities. Notably, PHD2 has the most charged or polar loop of the three. Additionally, Asp250 in PHD2 aligns with Ser234 in PHD1 and His72 in PHD3 has previously been reported.^7^ This suggests that the β2β3 loop of PHD2 could be the most solvent-exposed and may transition from a closed (as in PDB 3HQR) to an open conformation (PDB 3HQU) more readily than its counterparts in PHD1 and PHD3.

Even if dioxygen does not consistently diffuse through this loop, the sequence still forms a hydrophobic cavity in three-dimensional space, primarily shaped by residues Gly238, Leu240, Ile251, and the aliphatic portion of the Arg252 side chain. Just below this hydrophobic cavity lies Pro564. Many dioxygen-entry events from pathways P_m_ and P_4_ linger in this region under the β2β3 loop before dioxygen proceeds into deeper cavities within the catalytic cupin fold or exits back into the solution (Table 5; compare P_2_ column to P_4_).

Finally, the least frequently used access pathway, P_1_, showed the lowest relative occurrence specifically in the O_2_QD-parametrized simulations (Table 2). However, this pathway also included the longest observed residence time within a single hydrophobic cavity, approximately 330 ns, longer than any other access event. This extended residence occurred only because dioxygen was able to transition into the deep hydrophobic cavity of the P_m_ path by crossing a conceptual plane intersecting P_4_/P_1_, passing through Fe^2+^ and parallel to the axis of the cupin barrel.

The initial hydrophobic cavity along the P_1_ pathway is located close to the protein surface, at the PHD2 solution interface. This region forms what could be described as a convex surface cavity (CSC), composed of Gly253, Gly309, and hydrophobic side chains from Tyr303, Tyr310, and a methyl group from Thr308 (Figure 3). While this surface cavity may not be particularly significant in terms of kinetic trapping, the deeper hydrophobic cavities near the catalytic site are more interesting functionally.

Three primary hydrophobic cavities were identified deeper inside the cupin fold, on the opposite side from the deep P_m_ pathway cavity (Figure 5). The residues forming these cavities mainly belong to the outer β-sheet of the cupin fold, which faces the solvent but orients inward toward the Fe^2+^ center: The first cavity, frequently accessed during P4 access events, is composed of the benzyl ring of Tyr310, Pro564 from the substrate, and the methylene groups of Arg252. The second cavity is located adjacent to the catalytic triad that coordinates Fe^2+^, formed by His313, His374, Tyr310, and Val376. And finally, the third cavity is positioned above the catalytic site, deep within the central cupin fold, and potentially capable of sustaining long dioxygen residence times. This cavity is formed by Tyr303, Gly309, Gly341, Val376, Pro378, Ala379, and the aliphatic groups of Arg383.

**Figure 5:**
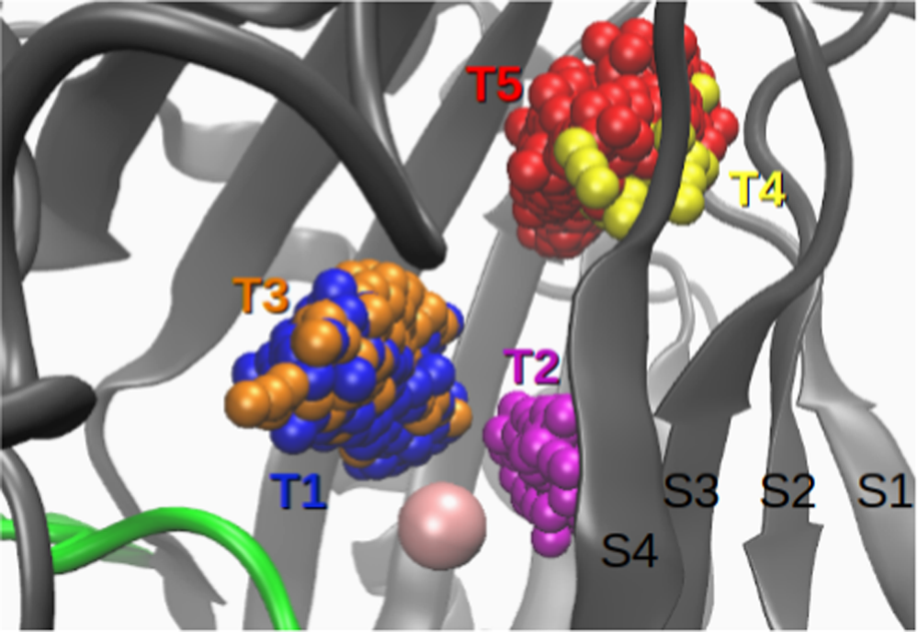
During a translocation event, dioxygen exhibits hydrophobic cavity hopping through the sequence T1 → T2 → T3 → T4 → T5, where each T represents a cluster occupying the visited cavity, and dioxygen is colored according to the cluster it belongs to. The metal is shown in van der Waals representation in pink.

### MSM Writing the Event States onto the PHD2 Structure

Mean first-passage times (MFPTs) were calculated for each system using reactive flux analysis from transition path theory (TPT), based on Markov state models (MSMs) constructed via maximum likelihood estimation (MLE).

For the O_2_QD simulations with 100 dioxygen molecules, 3498 MSM states were identified from 296 access events (Table 6). Reactive flux was computed using 301 unique end states, defined as frames in which dioxygen was within 6.7 Å of the Fe^2+^ atom, and 562 start states, defined as frames where dioxygen was approximately 22 Å from the Fe^2+^ atom, corresponding to positions outside the protein. For entry into the active site, these start states represent the first frames after dioxygen reached this outer distance; for exit from the active site, they correspond to the last frames before dioxygen reached it. The threshold used was chosen as a conservative approximation of the protein surface relative to the catalytic center, yielding a lower-bound estimate for MFPT. The resulting mean first-passage time was 1828 ns.

**Table 6:** Mean First Passage Times (MFPTs) from Reactive Flux Analysis Over the Entire Trajectory for Each System^a^

system	25_O2IF	50_O2IF	100_O2IF	100_O2QD
MFPT (ns)	2887	607	1310	1828
states	54,363	57,336	60,625	63,343
events	48	68	245	298

a Starting states were defined as frames where the average distance from Fe^2+^ exceeds 6.7 Å, and in the end states the average distance exceeds 6.7 Å, capturing potentially reversible events. A lag time of 100 ps was used. Reported values are in nanoseconds (ns), and the accompanying number of MSM states refers to the total number of states in the MSM, not the subset associated specifically with translocation events. The transition matrix was filtered to include only states during events in which dioxygen travelled from >22 Å to <6.7 Å relative to Fe^2+^. An event is defined when a dioxygen molecule reaches within 6.7 Å of Fe^2+^. The observed set of states corresponds to all occupied grid points containing dioxygen molecules, with a theoretical maximum of 64,000 states (40^3^ grid points). A cubic cutoff of 40 Å centered on Fe^2+^ was applied, such that molecules outside this cutoff were wrapped to the corresponding boundary of the simulation box along *X*, *Y*, and *Z* axes.

For the O_2_IF simulations containing 100 dioxygen molecules, 3270 states were identified from 245 events, comprising 291 end states (dioxygen within 6.7 Å of the Fe^2+^ atom) and 463 start states (dioxygen approximately 22 Å from Fe^2+^, outside the protein). These trajectories yielded a mean first-passage time (MFPT) of 1310 ns. In comparison, the O_2_IF simulations with 50 dioxygen molecules produced 1396 states from 68 events, including 119 end states and 134 start states, and resulted in a shorter MFPT of 607 ns (Table 6).

Interestingly, the mean first-passage time was shorter in simulations with 50 dioxygen molecules (607 ns) than in those with 100 molecules (1310 ns). This behavior likely reflects a reduction in pathway competition and crowding at lower dioxygen concentrations, allowing individual molecules to access the catalytic site more directly and efficiently. In contrast, higher concentrations increase the likelihood of transient interactions or steric hindrance near entry pathways, slightly delaying events. These observations highlight the strong influence of dioxygen concentration on translocation kinetics.

Notably, the MSM-derived MFPTs are consistent with manually tracked dioxygen translocation events using 10 ps frame resolution, although absolute time scales differ due to methodological differences. For O_2_IF with 100 molecules, a manual rate of 25 events per microsecond was observed compared to the MSM-derived MFPT of 1310 ns (one event every 40 ns for 100 molecules and one event every 4 μs for each molecule). Similarly, O_2_QD simulations yielded 17 events per microsecond (∼59 ns per event), compared to an MSM MFPT of 1828 ns.

To visualize the results from the MSMs, all states associated with access events were extracted, and the corresponding stationary distributions were mapped onto the equilibrated structures used in each simulation. Strong agreement was observed between these visualizations and the access pathways identified through both manual and computational trajectory analysis. In the O_2_IF set with 50 dioxygen molecules (Figure 6), density was observed along the primary pathway (P_m_), the route passing through the β2β3 loop that covers the Pro564 residue of HIF-1α and the fourth β-strand of cis-HIF-1α, labeled P_1_, and along the short α-helix at the N-terminus of the HIF-1α CODD 19-mer peptide (P_4_). In the simulation with 100 dioxygen molecules where more translocation events occurred, additional pathways were observed, including the β2β3 loop pathway of PHD2 (P_2_), an entry route along the anti-HIF-1α face of the cupin fold (P_r_), and a pathway beneath the HIF-1α CODD peptide between the two proteins (P_3_).

**Figure 6:**
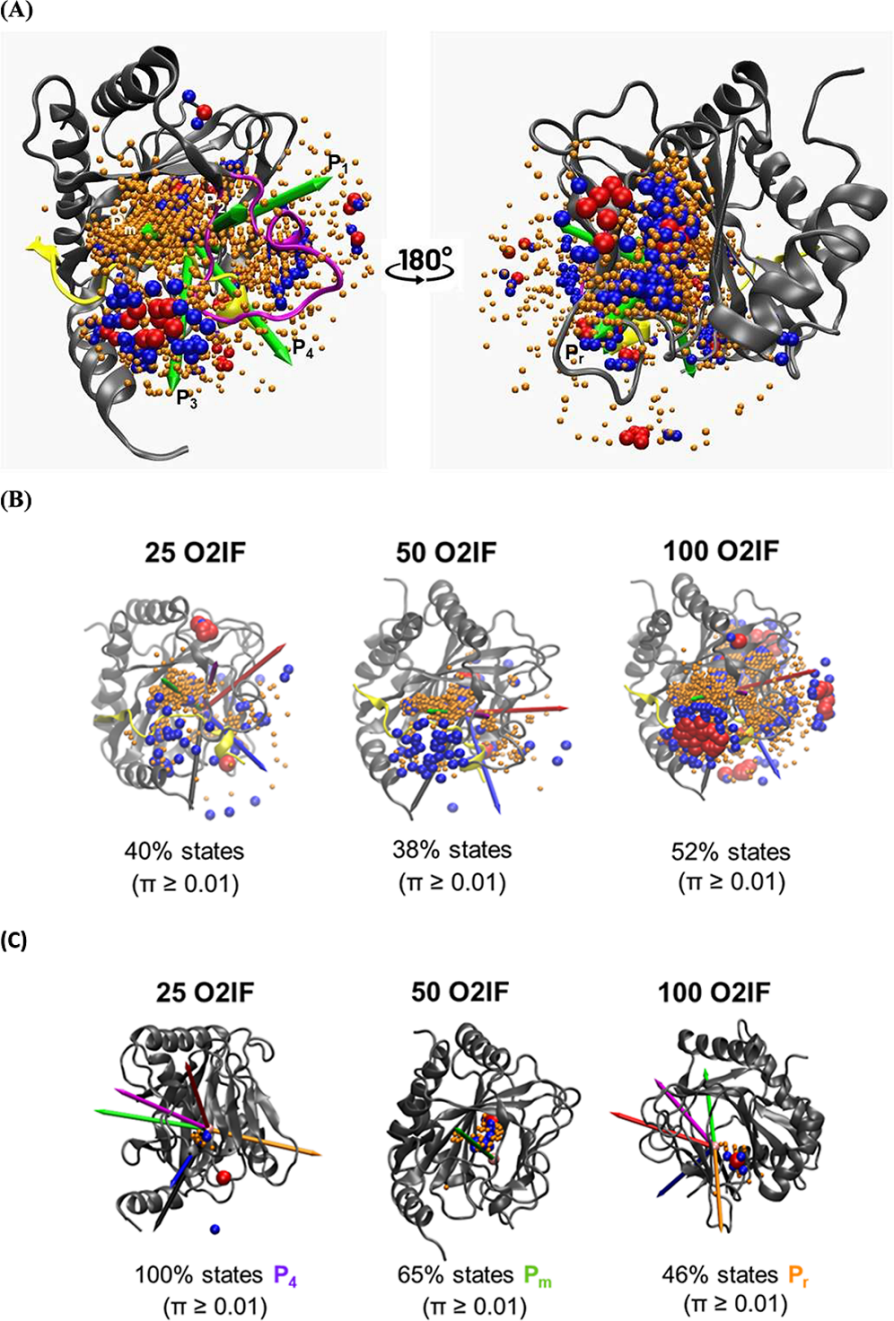
Normalized stationary probabilities overlaid with dioxygen pathways. (A) Left: Maximum-likelihood MSM stationary distributions overlaid on dioxygen trajectories in the O_2_IF system with 100 flooded dioxygen molecules. PHD2 is shown in gray cartoon representation, and the HIF-1α CODD peptide in yellow. MSM states are displayed to a radial distance of 20 Å from Fe^2+^ and are colored by normalized stationary probabilities calculated from dynamic states only: π > 0.01 in orange, π > 0.10 in blue, and π > 0.40 in red. States with π < 0.01 are excluded to improve visualization of Fe^2+^ and pathway arrows (green), which originate at the iron center. Right: View rotated by 180° to show the reverse face of the cupin fold, highlighting higher-probability (red) states at the top compared with the bottom of the reverse entry path. (B) Probability distributions of 25, 50, and 100 O_2_IF molecules relative to key protein positions (gray). (C) Selected examples of dioxygen translocation events for each system, colored and sized as stated above.

For the O_2_IF system with 50 dioxygen molecules, MSM states associated with dioxygen translocation events were visualized out to a maximum distance of 20 Å from the Fe^2+^ center. States were colored according to their normalized stationary probabilities derived from the MSM: orange for π > 0.01, blue for π > 0.10, and red for π > 0.40. States with π < 0.01 were excluded to improve the clarity of the visualization around Fe^2+^, the origin of the green pathway arrows (Figure 6). In total, 523 states (spheres) are shown (Figure 6), filtered from 1396 states associated with dioxygen translocation events. Regions of high MSM density were also observed from the O_2_QD simulation with 100 dioxygen molecules. For the O_2_IF system with 100 flooded dioxygen molecules, pathways for P_m_, P_r_, P_1_, P_2_, P_3_, and P_4_ can be identified from visualization of MSMs state probabilities onto PHD2. High-probability regions (red/blue) are located just below P_m_ (green arrow) and above HIF-1α (yellow), corresponding to the dominant starting pathway of P_m_. Additional high-probability regions appear to the right of P_1_ and P_4_, with the blue probabilities dipping toward the P_4_ vector. This indicates that the pathway traverses P_4_ more frequently than P_1_.

## Discussion and Conclusions

Multiple pathways from solvent-exposed regions through the cupin fold and into proximity (within 6 Å) of the Fe^2+^ catalytic center have been observed in our simulations of PHD2. These events can occur fast, with dioxygen traveling from 20 Å to less than 6 Å from Fe^2+^ (or in the reverse direction) within 50–60 ps, completing an entire diffuse-in/diffuse-out cycle in approximately 250 ps. Alternatively, dioxygen can exhibit long residency times within internal hydrophobic cavities, resulting in transport events occurring over much longer time scales, on the order of 250–300 ns.

A key question that emerges is how these hydrophobic cavities relate to the relatively slow catalytic rates of PHD enzymes.^18,19^ One hypothesis is that residence of dioxygen within these cavities delays coordination with the HXD triad, 2OG, Fe^2+^, and the hydroxylation target, Pro564, thereby limiting catalytic efficiency. Conversely, one could argue that these cavities provide a retention mechanism that prevents premature escape of dioxygen from the cupin fold before catalysis occurs. For example, dioxygen may enter an internal hydrophobic cavity located 5–8 Å from Fe^2+^, transiently exit, and subsequently re-enter the cavity, thereby increasing the likelihood of eventual engagement with the catalytic site, a “wash (escape), rinse (attempt catalysis), repeat (return to cavity)” dynamic. While this latter mechanism is less likely to accelerate catalytic turnover, it remains plausible as a means of increasing dioxygen availability near the active site.

Supporting the first hypothesis, our analysis identifies a set of hydrophobic residues contributing to hydrophobic cavities that, to date, have not been extensively investigated through mutagenesis. Most existing in vitro and *in vivo* studies have focused on mutations within the coordinating triad (e.g., H374R), charged residues in the β2β3 loop (e.g., R252A, D254A/H), the seven cysteines of the catalytic domain, P317R, R371H, and R383A.^24,41–44^ These mutations generally reduce catalytic activity or disrupt VHL association, indirectly inferring catalytic impairment, except for cysteine mutations, which showed no significant effect on P564 hydroxylation.

Notably, the first study to mutate hydrophobic residues W258F and W389F demonstrated a remarkable ∼10-fold increase in catalytic activity.^17^ This observation is consistent with the notion that the hydrophobic environment near the dioxygen pathways can exert kinetic control over dioxygen accessibility and residence near the catalytic Fe^2+^ center. Our data suggest that subtle modifications to these residues, especially those defining hydrophobic cavities, could alter local tunnel geometry, modulate transient dioxygen retention, and thereby influence catalytic probability.

However, caution is warranted, as many of these hydrophobic residues point toward the interior of the cupin fold, and mutating them could disrupt domain stability or folding, potentially resulting in protein degradation in vivo. Many such residues are small and aliphatic (e.g., Ala301, Ala385, Ala379, Gly341, Gly309, Val376), though some larger hydrophobic residues present more feasible mutation targets (e.g., Val376, Ile345, Ile207, Ile327, Leu343, Leu364, Phe366). Of particular interest are two tyrosine residues, Tyr303 and Tyr310, which are associated with hydrophobic cavity formation and are located nearest to Fe^2+^. Tyr310, situated on the final β-strand adjacent to the β2β3 loop, may play a role in loop opening, solvent accessibility, protein interactions, or hydrogen bonding with HIF-1α residues.^16^ Additional uncharacterized hydrophobic residues of interest include Gly238, Leu240, and Ile251 in the β2β3 loop; Ile256 near the P_m_ vortex; Phe391 within the P_3_ detour cavity; and the backbone of Phe378 (outside beta-sheet near red/yellow spheres in Figure 5).

From a kinetic perspective, the observed nonmonotonic dependence of mean first-passage times (MFPTs) on dioxygen concentration indicates that dioxygen entry into PHD2 does not scale linearly with molecular availability. The MFPT was longest at 25 dioxygen molecules (2123 ns), shortest at 50 molecules (607 ns), and increased again at 100 molecules (1310 ns), suggesting that dioxygen transport reflects a balance between encounter frequency and local crowding effects. At low concentrations, limited encounter probability may slow entry, whereas at high concentrations, transient interactions or steric congestion near the tunnel entrances can impede efficient access.

From a methodological viewpoint, although absolute MFPT values obtained are model-dependent, the observed nonmonotonic dependence of MFPT on dioxygen concentration and the pathway-specific differences in residence times indicate that dioxygen delivery to the PHD2 active site is governed not only by overall availability but also by local tunnel architecture and transient crowding, factors that could modulate enzymatic responsiveness to changing cellular dioxygen levels.

Beyond PHD2, the mechanistic insights uncovered here have broader implications for catalytic design and biotechnological applications involving metalloenzymes and oxygenases. Many catalytic systems, from nonheme iron enzymes to synthetic metalloproteins and nanocatalysts, depend on the efficient and selective delivery of small gaseous substrates to buried active sites. The demonstration that internal hydrophobic cavities can transiently retain dioxygen and regulate its local availability provides a transferable framework for understanding and engineering substrate transport in such systems. By applying similar long-time scale simulation and analysis approaches, it may be possible to rationally modulate tunnel geometry, hydrophobic character, or gating dynamics to optimize substrate access, turnover frequency, and oxygen utilization efficiency. These principles could inform the design of biocatalysts with improved oxygen sensitivity, stability under low-oxygen conditions, or enhanced catalytic performance in industrial and biomedical contexts.
